# A portable system for economical nucleic acid amplification testing

**DOI:** 10.3389/fbioe.2023.1214624

**Published:** 2023-08-02

**Authors:** Hui Dong, Jin Mo, Yongjian Yu, Wantao Xie, Jianping Zheng, Chao Jia

**Affiliations:** ^1^ School of Mechanical Engineering and Automation, Fuzhou University, Fuzhou, China; ^2^ Fujian Provincial Collaborative Innovation Center of High-End Equipment Manufacturing, Fuzhou, China; ^3^ Fujian Provincial Hospital, Fuzhou, Fujian, China

**Keywords:** paper microfluidics, POCT, NAAT, SARS-CoV-2 template detection, automatic image processing

## Abstract

**Introduction:** Regular and rapid large-scale screening for pathogens is crucial for controlling pandemics like Coronavirus Disease 2019 (COVID-19). In this study, we present the development of a digital point-of-care testing (POCT) system utilizing microfluidic paper-based analytical devices (μPADs) for the detection of SARS-CoV-2 gene fragments. The system incorporates temperature tuning and fluorescent detection components, along with intelligent and autonomous image acquisition and self-recognition programs.

**Methods:** The developed POCT system is based on the nucleic acid amplification test (NAAT), a well-established molecular biology technique for detecting and amplifying nucleic acids. We successfully detected artificially synthesized SARS-CoV-2 gene fragments, namely ORF1ab gene, N gene, and E gene, with minimal reagent consumption of only 2.2 μL per readout, representing a mere 11% of the requirements of conventional in-tube methods. The power dissipation of the system was low, at 6.4 W.

**Results:** Our testing results demonstrated that the proposed approach achieved a limit of detection of 1000 copies/mL, which is equivalent to detecting 1 copy or a single RNA template per reaction. By employing standard curve analysis, the quantity of the target templates can be accurately determined.

**Conclusion:** The developed digital POCT system shows great promise for rapid and reliable detection of SARS-CoV-2 gene fragments, offering a cost-effective and efficient solution for controlling pandemics. Its compatibility with other diagnostic techniques and low reagent consumption make it a viable option to enhance healthcare in resource-limited areas.

## 1 Introduction

According to the World Health Organization (WHO), there were 270 disease outbreaks worldwide from January 2019 to April 2023 (World Health Organization, 2022), including COVID-19, yellow fever, plague, and Middle East respiratory syndrome. COVID-19 has had an unprecedented social impact, and rapid and accurate detection of severe acute respiratory syndrome coronavirus 2 (SARS-CoV-2) is the first step in managing the pandemic (Tahamtan and Ardebili, 2020). However, global health inequities persist, with millions of people lacking economic opportunities, access to healthcare, and education (World Bank Group, 2020). Approximately 100 million people currently live in extreme poverty, with little or no medical protection. Molecular and serological methods are commonly used to detect and identify viruses. As a typical molecular biology technique for detecting and amplifying nucleic acids, nucleic acid amplification testing (NAAT) is able to amplify a specific region of the nucleic acid sequence, making it possible to detect very small amounts of the target material (Tarim et al., 2021). NAATs have been widely used in clinical settings for detecting various infectious agents, including viruses, bacteria, and parasites. Real-time reverse transcription polymerase chain reaction (RT-qPCR) is one of the most commonly used NAATs, with high sensitivity and specificity, and is recommended by WHO as the gold standard for diagnosing SARS-CoV-2 (Krsak et al., 2020). Since February 2020, PCR testing infrastructure has been widely built, and test results have become a critical criterion for managing personnel mobility, such as entry–exit control and work resumption. However, traditional RT-qPCR systems have much potential for improvement (Lin et al., 2022). Conventional RT-qPCR processes usually take at least 6 h from sampling to report printing. Commercial PCR instruments are expensive, have high energy consumption, require complex maintenance, and need professional technicians to operate (Tarim et al., 2023a). For remote and medically resource-limited areas, sample transportation and testing timeliness can have adverse effects on epidemic prevention and control. Loop-mediated isothermal amplification (LAMP) is an alternative method for nucleic acid testing. Unlike PCR, LAMP only needs a consistent temperature of 60–65°C, reducing the complexity of operating skills and instrumentation (Thompson and Lei, 2020). LAMP can also use bioluminescence to detect a pathogen, followed by real-time amplification analysis of the target during the reaction (Tarim et al., 2023b). LAMP kits for SARS-CoV-2 detection have been developed by Eiken Chemical.

Microfluidics technology allows for precise small-scale control and manipulation of fluids, enabling the integration of laboratory functions onto a single device to achieve accurate, sensitive, automated, and portable assays (Whitesides, 2006; Sun et al., 2020). Microfluidic paper-based analytical devices (µPADs), a branch of microfluidics, consist of hydrophilic cellulose or nitrocellulose fibers that transport fluids through a porous medium via capillary action (Martinez et al., 2010; Jia et al., 2020). Compared to conventional microfluidic devices, µPADs have several advantages, including easier fabrication, lower cost, easier disposal, and independence from pumps or other specialized equipment (Yang Ruey-Jen et al., 2018; Sun et al., 2019). The most attractive application of µPADs is in the development of point-of-care testing (POCT) platforms, which may minimize the need for sophisticated and laboratory-based analytical procedures (Wei et al., 2016). Recently, genetic materials of pathogens such as *Staphylococcus aureus* (Chi et al., 2018), *Plasmodium* (Xu et al., 2016; Reboud et al., 2019), bovine herpes virus-1 (Yang Zhugen et al., 2018), *Neisseria meningitidis* (Dou et al., 2017a), *Streptococcus pneumoniae* (Dou et al., 2017b), hepatitis C virus (McConnell Weronika et al., 2021), vector-borne viruses (Yao et al., 2021), and *Vibrio parahaemolyticus* (Zhang et al., 2021) have been detected by LAMP or PCR in µPADs. Paper-based LAMP or PCR for SARS-CoV-2 detection may provide novel population screening and POCT tools as clinically relevant concentrations of SARS-CoV-2-specific sequences have been measured in paper microfluidics (Hui et al., 2020; Sreejith et al., 2021).

We have previously validated the detection of SARS-CoV-2 using the LAMP method in paper units (Sun et al., 2023) and a commercial on-chip reaction apparatus for rapid diagnosis of COVID-19 (Sun et al., 2021). In this study, we improved the chip design and fabrication process, and we developed a novel instrument system for the portable intelligent diagnosis of COVID-19. The system integrates modules for intelligent temperature control, fluorescence signal detection, intelligent identification of reaction units, and digital image analysis. We used artificially synthesized SARS-CoV-2 gene fragments (ORF1ab gene, N gene, and E gene) as targeted samples. Laser cutting, thermal lamination, and additive manufacturing were employed to fabricate the µPADs and frame of the portable system. The chip cost was only about $0.30, and the total cost of the system was approximately $160 (Supplementary Table S1). The total reagent consumption for each readout was 2.2 μL, which is only 11% of the requirements of conventional in-tube methods. Considering both the expense of commercial reagents and the cost of chips, the approximate cost per test is $2.10. The power dissipation of the system was low at 6.3 W, accounting for only 1.05% of the energy used by traditional instruments. Dilution testing results showed that the equivalent limit of detection (LOD) was 2.2 copies of RNA template. In addition, the detection throughput can be customized and expanded. Its power and reagent consumption are lower than most existing devices, making it ideal for improving community medical care and family health protection. The approach is compatible with other advanced molecular biology techniques and could help promote POCT system development. It can potentially be deployed in remote or resource-limited regions for infectious disease diagnosis.

## 2 Materials and methods

### 2.1 Principle and materials

Thanks to the operating simplicity and no requirement of thermal cycling, RT-LAMP is very suitable for on-chip tests and was adopted for the proof-of-concept study. Briefly, a typical LAMP reaction employed four types of primers complementary with six distinct regions of the target gene (Tomita et al., 2008). By placing the mixture of targeted templates, primers, dNTPs, Bst polymerase, and fluorescence dye at a consistent temperature (60°C–65°C), amplification can be continued and cycled. To realize the real-time data collection and quantitative analysis, LAMP employed calcein as the fluorescent indicator to measure the accumulative amplification products. Calcein was first bonded to manganese ions, resulting in fluorescence quenching. With amplification processing, pyrophosphate ions were produced as a by-product of dNTPs. Manganous ions were then deprived of calcein by the generated pyrophosphate ion, causing fluorescence emission. By exciting at 480 nm, the fluorescence spectra of the LAMP reaction solution peaks around a wavelength of 515 nm. We selected a RT-LAMP kit (Loopamp catalog#: 03001) manufactured by Eiken Chemical Co., Ltd. (Tokyo, Japan). Operating procedures followed the manufacturer's manual.

### 2.2 Device design and fabrication

**FIGURE 1:**
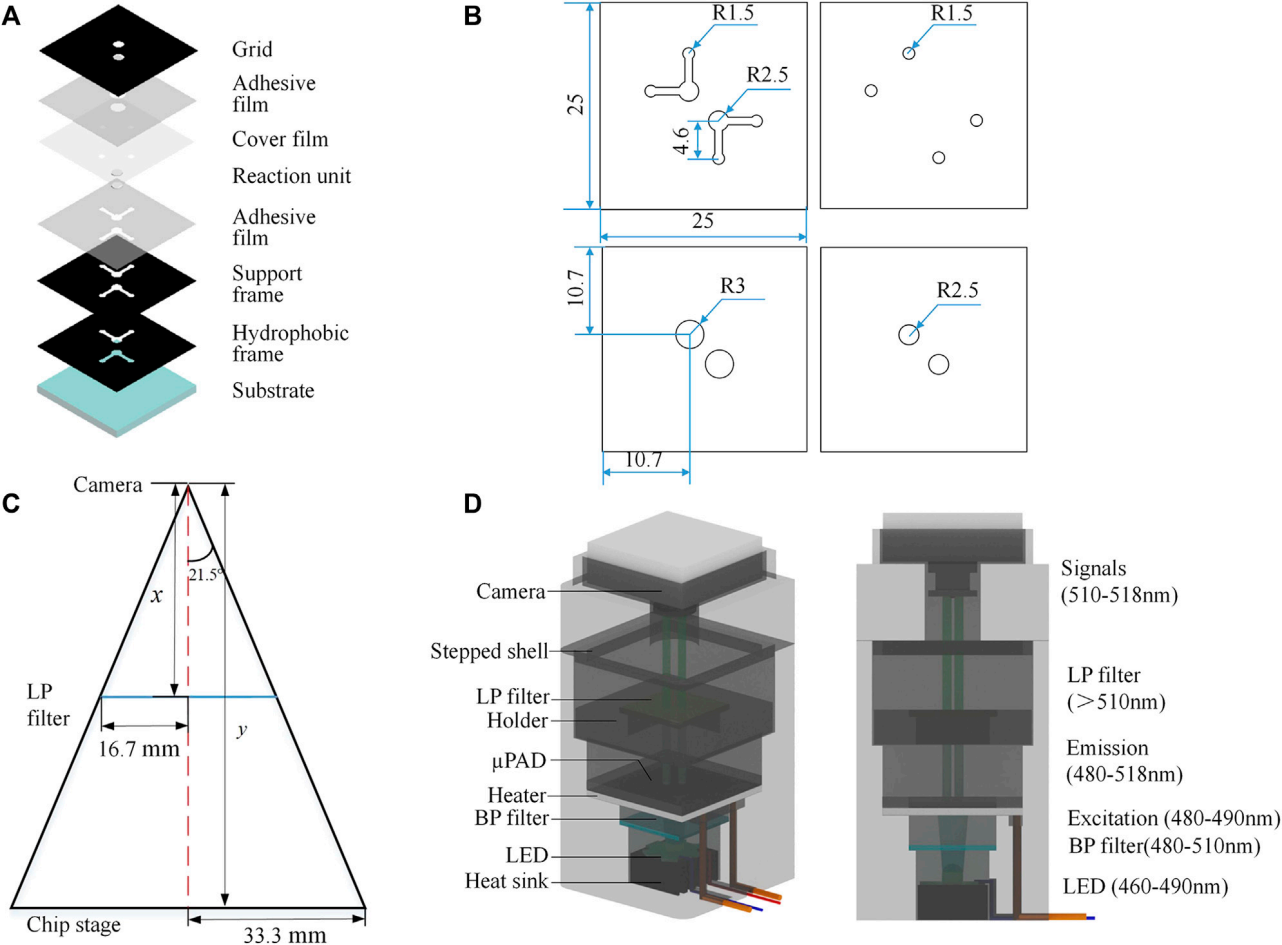
Principle and design. **(A)** Multilayer µPAD design. **(B)** Position and size of the pattern (unit: mm). **(C)** Geometric design of the detection field. **(D)** System design.

Multi-layer µPADs were designed and fabricated using the capillary self-driving properties of cellulose materials (Figure 1A). The device enabled the micro-injection, diffusion, and storage of liquid samples without any external pumping. Reagents could freely flow to the reaction area for LAMP testing. The device consisted of substrate, hydrophobic and support frames, two reaction units, cover film, two adhesive films, and a detection grid, aligned and stacked from bottom to top. The materials employed in the chip are listed in Supplementary Table S2. The substrate measuring 25 × 25 mm was made of high-purity quartz glass, which offers strong chemical inertness, fine thermal conductivity, and high transparency; it efficiently transferred the heat required for the LAMP reaction. The hydrophobic frame was made of black polyvinyl chloride (PVC) and connected to the glass substrate using 0.1-mm-thick pressure adhesive film. This frame created hydrophobic channels which allowed reagents to flow smoothly into the reaction area. The reaction unit layer adopted a hollow design with two symmetrical right-angle patterns, each including a pair of sample inlet/outlet and detection units. The paper-based reaction unit employed Whatman grade 1 filter paper, which had uniform properties, moderate fluid flow rate, and liquid absorption capacity, as well as fine chemical inertness and biocompatibility. The support frame not only increased the channel height but also served to fix and connect the upper and lower layers. Similarly, the adhesive film maintained the stability of the chip structure. The cover film, made of 0.03-mm-thick PVC, functioned to inhibit reagent evaporation during the reaction process. The detection grid, made of black PVC adhesive, directly covered the chip, exposing only the two circular detection areas and reducing interference from surrounding stray light. The chip channel design and schematic diagram of sample loading is shown in Supplementary Figure S1. The layout design and the shaping of PVC, PET, and filter paper materials were completed using Adobe Illustrator software. Images were generated at 300 ppi precision for import into a laser engraving machine. The glass substrate was pre-cut by the manufacturer. After accurately aligning and assembling the quartz glass substrate with the pre-processed hydrophobic frame, supporting frame, paper-based reaction unit, and cover film, the multi-layer structure was molded by a drum-type plastic molding machine. At this point, the fabrication of the chip was accomplished. Overall, the fabrication and packaging procedures of the device were simple and fast.

### 2.3 System design and integration

The instrument system comprised a temperature control module and a fluorescent detection module, with the geometric design of the detection field shown in Figure 1C. The chip area was 25 × 25 mm^2^, and the detection zone was a circle with a diameter of 5 mm. We selected a camera with a focal length of 8 mm and a viewing angle of 43°, which could be manually adjusted to meet the imaging requirements. The calculated object distance was 63.25 mm—we used 65.0 mm in practice, which proved sufficient for our needs. The temperature control module utilized a 40 × 40-mm square ceramic heating plate (metal ceramics heater, MCH) as the heating device, with a circular through hole of 9 mm diameter in the center of the plate for direct excitation light source irradiation to the reaction zone. The ceramic heating element was installed on the middle platform of the shell and connected to a DC 5.0 V lithium battery with a power consumption of 4.0 W. Figure 1D shows the system design.

**FIGURE 2:**
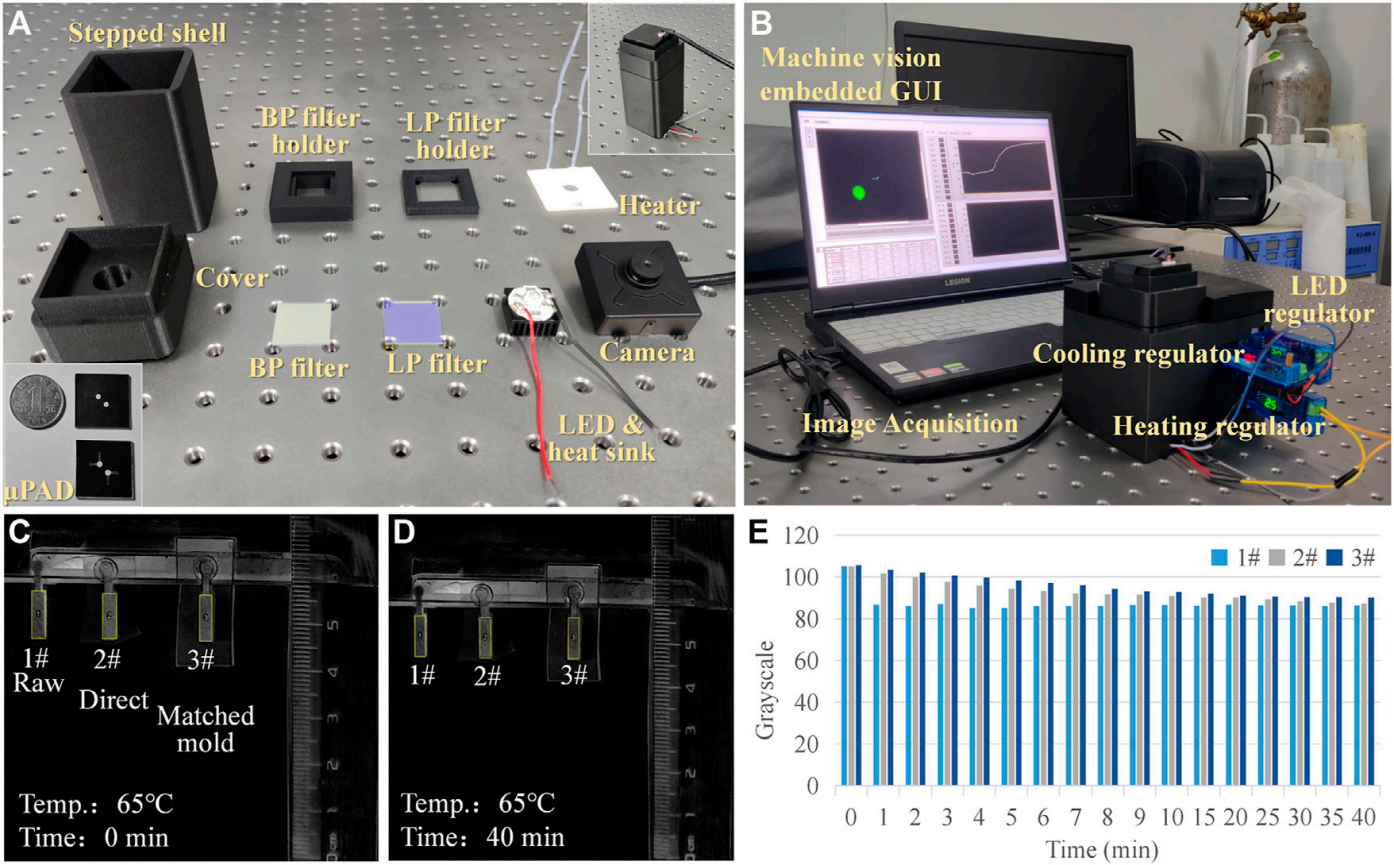
Prototype and evaporation testing. **(A)** Components of the portable system. **(B)** Packaged prototype. **(C,D)** Comparison of different evaporation restricting treatment methods. **(E)** Grayscale analysis of the evaporation testing images.

The optical module adopted a parallel optical path design (Figure 1D). The LED light source, band-pass (BP) filter, long-pass (LP) filter, chip detection area, and CMOS camera are arranged in parallel from bottom to top. According to the LAMP detection principle, the maximum excitation wavelength of calcein was 497 nm and the maximum emission wavelength was 518 nm. Here, an LED light-emitting chip with an emission wavelength of 460–490 nm was used in the center of the rice-shaped circuit board as the excitation light source. A BP filter (wavelength: 480–510 nm) was directly installed above the light source to improve the monochromaticity of the excitation light. A LP filter (wavelength>510 nm) was installed above the detection area of the paper chip to improve the signal-to-noise ratio. The CMOS camera (12 million pixels) was located above the LP filter, equipped with an 8-mm focal length lens and a lens angle of 43°; it could capture clear images within 70 mm. Fluorescent images reflecting an on-chip reaction were transferred to computer via USB communication. Prototypes of the devices, hardware modules, and the packaged system are shown in Figures 2A, B. The power consumption of the components and the whole system was low (Table 1). The most attractive advantage of POCT devices lies in their ability to perform real-time diagnosis near the patient. The turnaround time can be significantly reduced by eliminating sample transportation.

**TABLE 1:** Calculation and comparison of power consumption.

Developed system		Commercial instrument	
Component	Power(W)	Model	Power(W)
Blue light LED + cooler	1.0	Abbott^®^	950
Porcelain heater	4.1	m2000rt	
Cooling fan	0.5	Thermo Fisher^®^	500
CMOS HD camera	0.8	MiniAmp Plus	
**System total**	**6.4**	BIOER^®^	180
		FQD-16 A (Mini)	

The bold value means the total power of the proposed system.

### 2.4 Image processing software

The fluorescence image processing algorithm comprised two main parts: the upper computer control program using LABVIEW and the data treatment program using MATLAB. This software configuration harmoniously integrated the automatic measurement and instrument control abilities of LABVIEW with the data processing ability of MATLAB. The LABVIEW program was developed for image acquisition, image preprocessing, image circular area recognition, coordinate extraction, data storage, and visualization. Image acquisition relied on the VAS (Visual Acquisition Software) toolkit, and image processing and pattern recognition employed the VDM (Vision Development Module) toolkit. MATLAB performed grayscale image conversion on the input image, and the grayscale image was imported as an array of 8-bit unsigned integers ranging from 0 to 255. During detection, the center coordinates and radius of the fluorescent area identified by LABVIEW were input into MATLAB as parameters, and the average gray value of the designated circular detection area was then calculated. We set the image acquisition time interval to be 3 s (which could be adjusted down to 1.0 ms) and presented the calculated average gray value as a real-time waveform chart (i.e., amplification curve). By calling the MATLAB ActiveX interface, the two programs were connected.

## 3 Results and discussion

### 3.1 Evaporation

Reagent evaporation during thermal cycling affected the performance of on-chip amplification. The chip packaging technique was imported to inhibit evaporation. A comparative experiment was performed to test the effect of the lamination process on the evaporation restriction. The first group was the control using raw paper without any treatment; the second group used direct ordinary plastic encapsulation; the third group used a pairing mode of paper and plastic. The pairing mode referred to tightly embedding paper into a layer of plastic film with the same thickness of paper. The matched paper and film were then placed into the lamination machine synchronously. The process could minimize the edge gap caused by the thickness of the paper during lamination. Three groups were dropped into an identical volume of red ink and placed onto a heating plate with a constant temperature of 65°C, and the images representing changes in the samples over time were recorded (Figures 2C, D). Black and white inversion processing was performed, and ImageJ software was then employed to calculate the average grayscale value of the images at different times (Figure 2E). It was evident that the grayscale value of the raw group (1#) decreased rapidly. For the ordinary (2#) and pairing mold lamination (3#) groups, there was a difference in the decreasing speed of the grayscale values. A stable grayscale value meant that the concentration of the reagents was higher, and the solvent evaporation inhibition was better. After 1-min heating, the evaporated volumes were 34.6, 6.2, and 4.6 μL, and the corresponding evaporation loss rates were 17.3% (1#), 3.1% (2#), and 2.3% (3#). Therefore, the paring mold lamination was more suitable for on-chip testing. Based on the method proposed in this article, the relative evaporation of the reagent after heating for 40 min was 14.8%. Since a significant fluorescence reaction was observed on the chip, it could be inferred that the evaporation of the reagent did not affect the experimental results.

### 3.2 On-chip heat transfer

**FIGURE 3:**
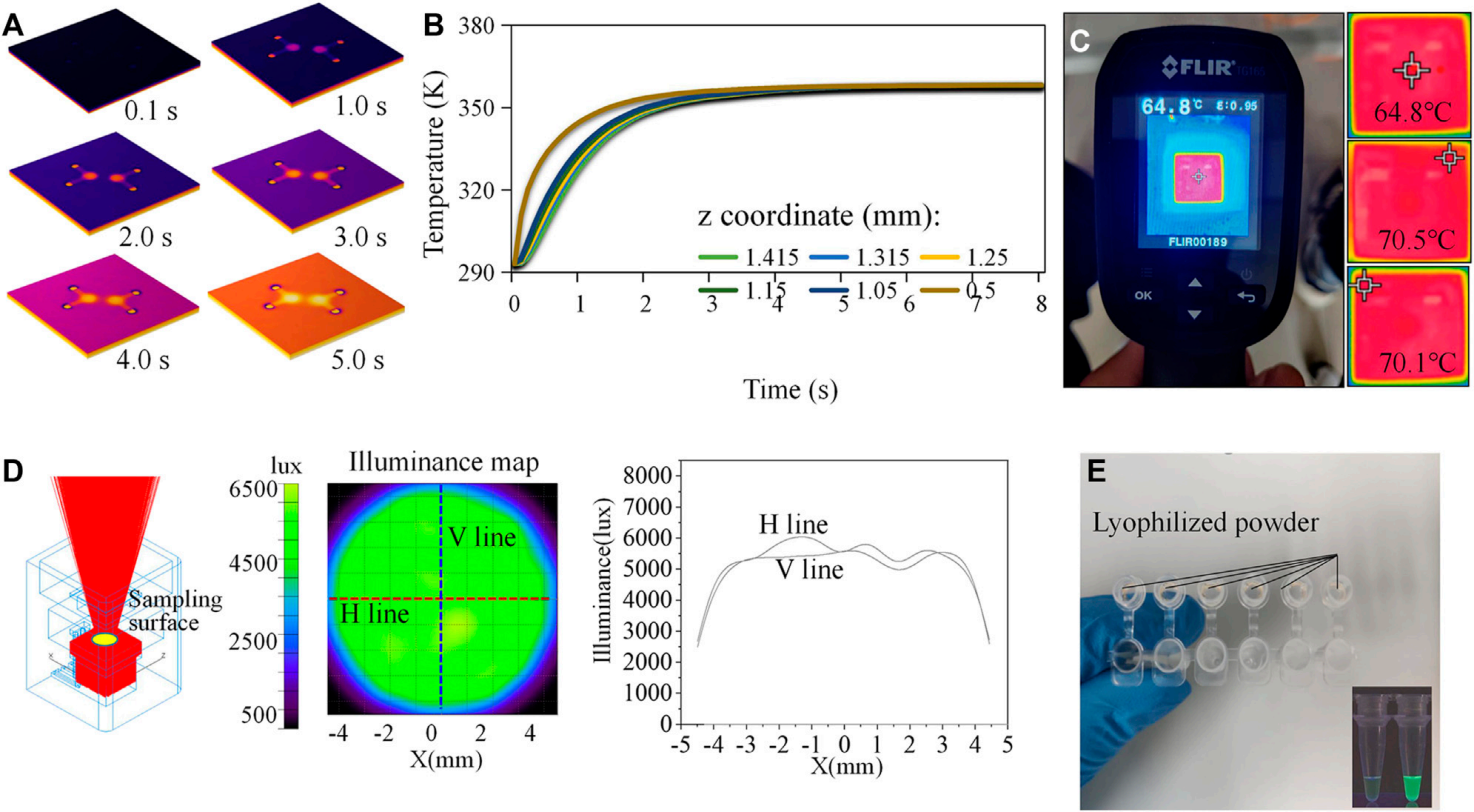
Simulation and calibration study. **(A)** Transient simulation of the on-chip heat transfer process. **(B)** Temperature distribution along the axis of the circular detection area. **(C)** Infrared thermal images reflecting the temperature distribution. **(D)** Simulation of the light trace on the chip surface. **(E)** In-tube tests.

Since the MCH with a hollow circle was employed for on-chip thermal control, the center of the paper-based chip was not in direct contact with the ceramic heating sheet. Heating of the reaction zone mainly relied on the fine thermal conductivity of the quartz glass substrate. In parallel, the resistance of the heating element was relatively small (∼0.7Ω). If the power was too high, the chip overheated, and the heating element could be damaged. The rated voltage of the selected lithium battery was 5.0 V, and the maximum current was 2.4 A. In order to stabilize the temperature of the chip reaction area in the range of 60–65°C, a voltage regulator was needed to adjust the input voltage and current. Numerical simulation in COMSOL Multiphysics software was employed to model and simulate the on-chip heat transfer process to optimize the working parameters of the MCH. The quantitative relationship between the temperature distribution of the heating element and the temperature of the reaction zone was obtained. The heat transfer in the solids and electromagnetic modules of the software were coupled. The geometric parameters followed 1:1 mapping of the size of the MCH and the chip. Due to the simple structure of the model, a tetrahedral structure containing 51796 elements was used for meshing. The minimum element quality was 0.1768, and the average quality was 0.657. According to the results in Figure 3A, when the temperature of the ceramic heating element reached 70°C (343.15 K), the temperature on the chip reaction zone was 65°C (338.15 K), which can meet the LAMP temperature requirements. In addition, the temperature distribution along the axis of the circular detection area indicated that the heat transfer process was rapid and efficient (Figure 3B). The MCH took only about 5 s to heat the device from room temperature to 65°C. We recorded the working parameters (boundary conditions) as voltage 1.7 V and current 2.4 A. An infrared thermal imager was used to experimentally measure the temperature of the reaction zone of the chip and the temperature distribution of the MCH (Figure 3C). As a reference, we also measured the temperature of the MCH via a thermocouple thermometer under the same condition (Supplementary Figure S2). Within the enclosed space of the 3D-printed structure, the heat dissipation layer blocked the heat exchange between the internal and external environments of the instrument. The heating element had a temperature sensor chip that measured the temperature, and the output current was controlled by a PID (proportional-integral-derivative) algorithm. The current was adjusted based on the actual temperature of the chip. These two factors ensured that the temperature of the chip was effectively maintained at a constant value. Using the voltage and current obtained by the simulation, the edge temperature of the MCH was about 70°C and the center temperature of the chip was 64.8°C. The measured results were consistent with the simulation. Furthermore, the voltage regulator was equipped with an active cooling fan with a power of 0.5 W, and thus, the total power consumption of the heating module was as low as 4.58 W.

### 3.3 Illumination

The most commonly used detection optical paths were orthogonal, non-confocal, confocal, and parallel. The first three were relatively complex and reluctant to system miniaturization. The parallel optical path can make the structure more compact, but interference from stray light on the detection could usually not be neglected. To minimize this interference, we shielded all areas with an opaque PVC film excepting the reaction zone. We used TracePro optical simulation software to quantitatively evaluate the performance of illumination (Figure 3D). It is evident from the results that the light generated by the light source was emitted following the ideal trajectories. In addition, the illumination on the surface containing the reaction area was uniform, which was suitable for fluorescence detection. A spectrum analyzer (OHSP-300P) was used for experimental tests. The measured central wavelength of the light source was 463 nm, with an intensity value of 6.3 μW/cm^2^/nm. After the BP filter, the central wavelength of the light source was 479 nm, with an intensity value of 1.6 μW/cm^2^/nm. After the LP filter, the central wavelength of the light source was 509 nm, with an intensity of 0.04 μW/cm^2^/nm. The camera mainly collected fluorescence signals of wavelengths greater than 510 nm. Thus, the main fluorescent signals after LP filtering were the emission light reflected by calcein reporter. The total power of the optical module was 1.8 W.

### 3.4 Validation

**FIGURE 4:**
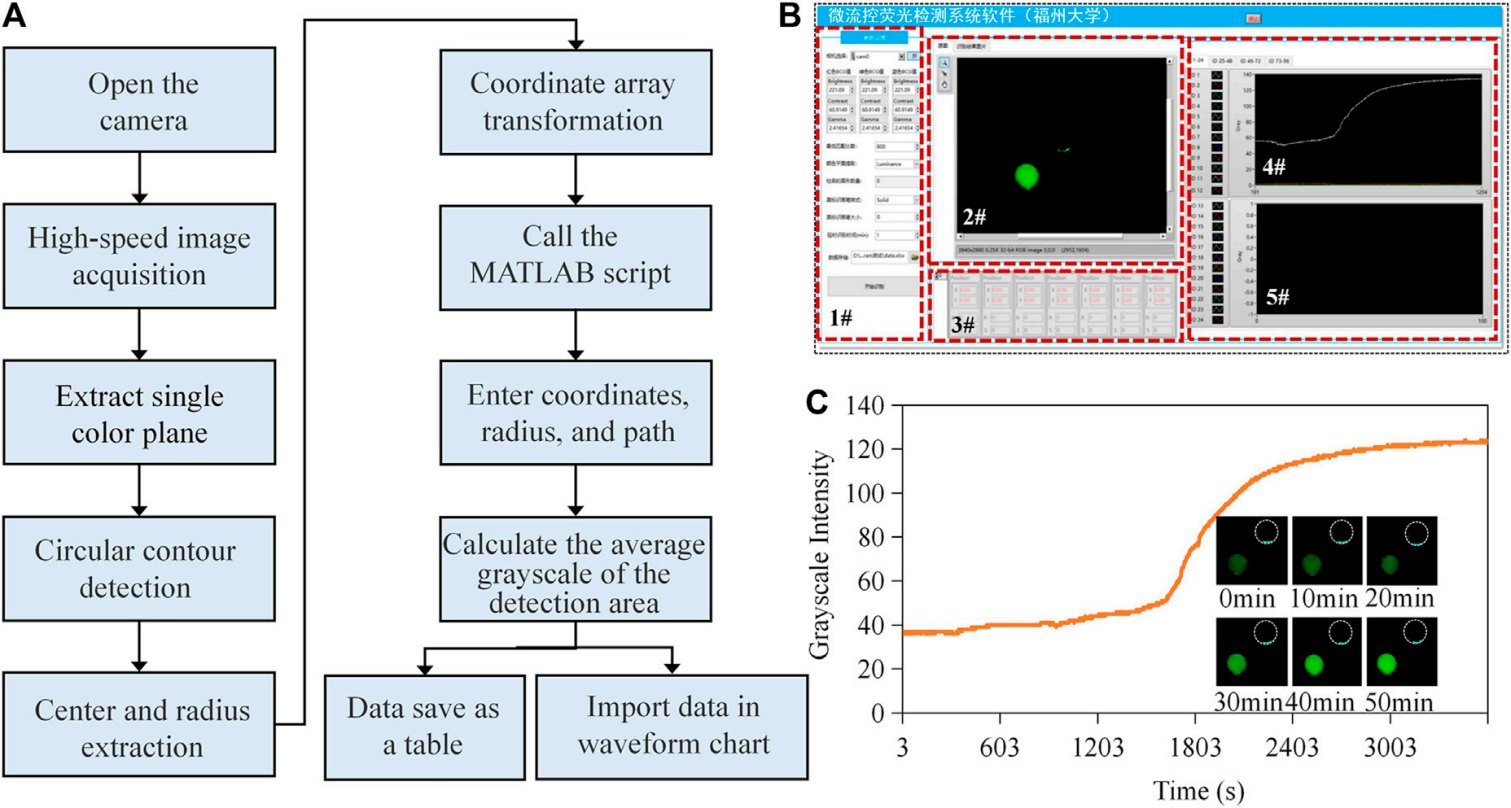
Workflow and validation test. **(A)** Operating procedure of the method. **(B)** Graphical user interface of the software application. Software is available at: https://drive.google.com/drive/folders/1QuO9x_L3MHk5wjNlSv4XmO3rJZdO7Jv3?usp=drive_link (**C**) Validation test of the real-time LAMP using paper microfluidics.

The preparation of reagents was conducted in a sterilized biological safety cabinet. We used a micro-pipette to measure 2.2 μL of the mixing reagent and transferred it to the input of a µPAD. For the negative control, we used the same volume of reagent without any target templates. We first conducted in-tube validation tests to verify the commercial kit containing artificially synthesized RNA templates of SARS-CoV-2 (Figure 3E). We then sealed the inlet and outlet with high-temperature-resistant PET pressure-sensitive adhesive and placed the device on the MCH to perform on-chip validation tests. The working flow of the test is shown in Figure 4A, and the chip reaction process is real-time presented on the GUI, as shown in Figure 4B. Window #1 on the panel was for parameter setting and selection. On-chip reactions were real-time visualized in Window #2. The brightness of the positive sample area (down left) of the chip increased after the reaction, while the brightness of the negative sample area (up right) remained unchanged. We set the image acquisition time interval to 3s and obtained the real-time average grayscale value of the detection area containing 11,310 pixels. The center ordinates and radii of the machine vision detected cycles were shown in Window #3, and the averaged grayscale values were plotted in Windows #4 and #5. The on-chip amplification curve typically included periods of baseline, exponential growth, linear growth, and plateau, and its shape was similar to a sigmoid curve. The results demonstrated that the proposed approach can effectively identify artificially synthesized SARS-CoV-2 samples. The amplification signals became distinguishable after about 28 min.

### 3.5 Repeatability

**FIGURE 5:**
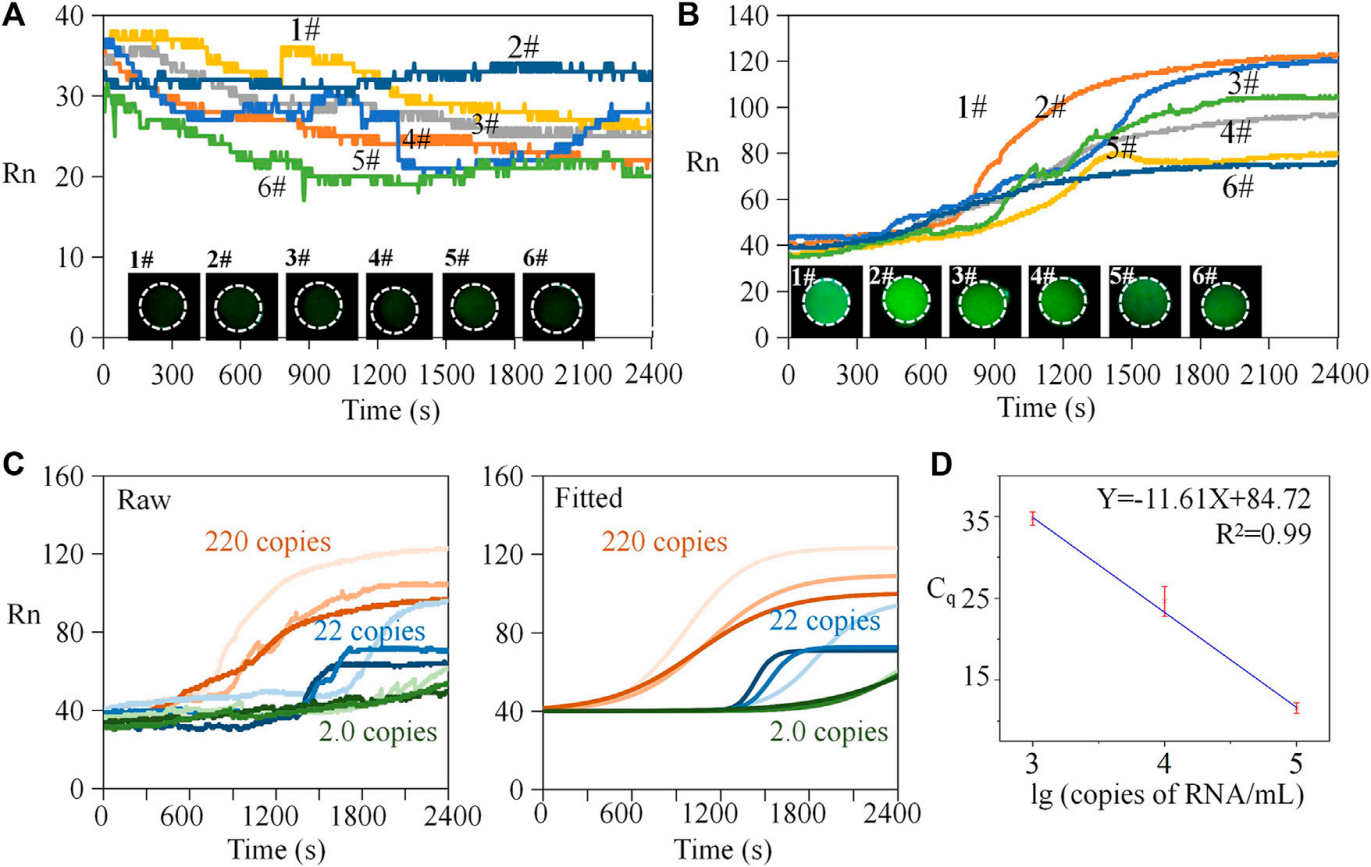
Repeated tests and standard curve analysis. **(A)** Repeated tests of negative samples. **(B)** Repeated tests of positive samples. **(C)** Raw and fitted amplification curves of diluted positive samples. **(D)** Standard curve analysis.

The workflow of the experiment is provided in Supplementary Table S3. The negative samples used the identical reagents without RNA templates, and the positive samples included RNA templates with a concentration of 10^5^ copies/mL. For negative sample testing, the results illustrate that the reaction without gene templates also had an irregular fluorescence intensity (Figure 5A). There was no obvious exponential, linear amplification period inside the curve, and the data were generally in a range of 25–50. The standard deviations (SDs) of the six negative sample tests of 3.54, 1.31, 2.16, 3.89 1.41, and 4.01 indicated that overall data fluctuation was small. If we compared the data on different groups at the same time, the mean SD value was 1.91. It can be concluded that the negative data were stable, and the systematic and random errors relatively small. Correspondingly, end-point fluorescent images of the six reactions were dark. For positive samples (Figure 5B), samples 1# and 2# were a homologous reagent which were from the same batch and in the same tube, while samples 3# to 6# used another homologous reagent. The overall trend of the amplification curves in each group was consistent, and the curve profile was sigmoid in shape. In each group, obvious amplification can be seen at the time of 20 min, and the final grayscale values were all above 60. Among these, Curve 1# showed obvious amplification at 15 min. Considering Curves 1# and 2#, the mean SD of the two groups at the same time was 5.18. Considering Curves 3# to 6#, the mean SD was 2.71. Therefore, the deviation within the homologous reagent group was low, indicating that systematic and random errors were small. We also put the data in a bar chart for illustration (Supplementary Figure S3). The negative control experiment demonstrated that the endpoint fluorescence image was relatively weak compared to the positive results. Figures 5A, B provide visual statistics indicating that the endpoint values of the amplification curve (<40) were significantly lower than those of the positive samples (>60). Consequently, it can be inferred that, even with only 2.2 copies, effective amplification occurred on the chip. Consequently, we established 2.2 copies as the detection limit for this experiment.

In addition, both negative and positive sample curves had local fluctuations. There were two potential reasons for this phenomenon. 1) Molecular motion: the thickness of the filter paper (0.2 mm) was much lower than the plane feature size (2.5 mm), and the molecular thermal motion of the reactants between the porous media was more active on the plane, which may result in more detectable signal fluctuations. 2) Acquisition frequency and raw data without preprocessing: the amplification curve recorded the fluorescence signal every 3 s without processing such as data homogenization, smoothing, and curve fitting. Indeed, traditional instruments often use these treatments for better visualization and interpretation.

### 3.6 Quantification

Establishing a standard curve is key to obtaining the absolute quantity of targeted templates. In PCR, reporter dyes are typically used to label the amplification process of the target sequence, while reference dyes are used to provide an internal reference and correct for experimental variations. Rn (normalized reporter signal) is obtained by normalizing the fluorescence signal of the reporter dye with the fluorescence signal of the reference dye, resulting in a normalized reporter signal value. The calculation of Rn value is determined thus:

$$Rn=\frac{F-F_{0}}{F_{\max}-F_{0}},$$

where F represents the fluorescence signal of the reporter dye in the current amplification cycle, F_0_ represents the fluorescence signal of the reference dye in the current amplification cycle, and F_max_ represents the maximum fluorescence signal of the reference dye. By standard curve analysis, the initial copy number of the template can be inferred from the value of quantification cycle (Cq). Cq represents the cycle number at which the fluorescence signal surpasses a predetermined threshold. Ten-fold gradient dilutions (i.e., from 1 × 10^3^ to 1 × 10^5^ copies/mL) were performed on the RNA templates with known concentrations for generating the standard curve. Amplification curves by raw data are shown in Figure 5C. Then, a Boltzmann function was used to fit the raw data thus:

$$y=\frac{A_{1}-A_{2}}{1+e^{\left(x-x_{0}\right)/dx}}+A_{2}.$$

Herein, y is the fluorescent intensity, A_1_ is the initial intensity, A_1_ is the end-point intensity, x is the reaction time, x0 is the time when the fluorescence intensity was (A_2_-A_1_)/2, and dx is the interval time step of fluorescence mutation. The fitted curves are presented in the right part of Figure 5C. Finally, we obtained the standard curve (Figure 5D). Using an identical threshold value, the mean of the Cq was 11.6, 24.6, and 34.8 for the positive samples containing 1 × 10^3^, 1 × 10^4^, and 1 × 10^5^ copies/mL templates. From the view of detection rapidness, the on-chip method presented detectable signals within 35 min—faster than many traditional in-tube tests and acceptable for POCT. From the perspective of detection sensitivity, we introduced 2.2 µL of mixed reagents containing 0.36 µL templates; therefore, the equivalent quantity of the detected template was only one copy (a round-off number). The limit of detection was 1 × 10^3^ copies/mL, equivalent to one copy or single RNA template per on-chip reaction. Based on the standard curve analysis, the quantity of the target templates can be determined.

### 3.7 Assessment

To evaluate the benefits of the paper-based device described in this article, we conducted a parallel experiment using a conventional tube-based approach (Supplementary Figure S4). The experiment was conducted using a LongGene^®^ Q2000B fluorescence quantitative PCR instrument, and it utilized identical reagents to those employed in the paper-based experiments, including positive samples and negative controls. By comparing the results of both methodologies, it was evident that paper-based detection was commendably efficient, with a clear amplification signal becoming visible within approximately 30 min—comparable to the traditional tube-based method. Additionally, the paper-based device requires a minimal reaction sample volume of only 2.2 μL per test, resulting in negligible reagent consumption. In contrast, conventional tube-based detection necessitates a reaction sample volume of 23 μL according to experimental standards, alongside the requirement for a high-powered instrument. Hence, the paper-based chip method offers the advantages of being environmentally friendly and cost-effective.

Moreover, we performed a comprehensive comparative analysis between the methodology outlined in this article and a range of alternative methods and studies. First, we compared the test results of our study with the published literature and the conventional PCR experimental data provided by instrument manufacturers. The LOD of different methods and instruments is shown in Table 2: our method has a lower limit of detection compared to existing PCR methods. We then conducted a comprehensive comparison of chip size, cost, power consumption, LOD, and detection time with other LAMP-based methods (Table 3). We found that the device in this study is simple and compact in structure, and the cost of chip fabrication and detection is relatively low. Additionally, the utilization of parallel light excitation pathways improved the visibility of the fluorescent signal, which significantly reduced the detection limit. However, this study’s current focus is microfluidic chips with two levels of throughput. Further design enhancements are required to enable high-throughput nucleic acid detection. Additionally, the experimental methods need optimization to further reduce the detection time.

**TABLE 2:** LOD of existing PCR instruments and methods.

Instrument model	Company	Limit of detection
ABI 7500 Fast	Applied Biosystems	61–96 copies
QuantStudio 5	Thermo Fisher Scientific	56–79 copies
LightCycler 480 II	Roche Diagnostics	62–89 copies
BD Max	Becton Dickinson and Company	61–96 copies
CFX96™	Bio-Rad Laboratories, Ltd	53–92 copies

Author	Publication	Limit of detection
Garg et al. (2021)	Journal of Medical Virology, 2021	20 copies
Alteri et al. (2020)	PloS one, 2020	2.9 copies
Pekosz et al. (2021)	Clinical Infectious Diseases, 2021	6 copies
Piana et al. (2021)	MSphere, 2021	5.2 copies
Chan et al. (2020)	Journal of Clinical Microbiology, 2020	11.2 copies
This work		2.2 copies

**TABLE 3:** Comparison between LAMP-based methods.

Work	Chip size	Cost	Power	LOD	Time (min)
Tarim et al. (2023b)	—	< $200	—	10 copies/μL	55
Yao et al. (2020)	d = 90 mm	—	—	50–100 copies	45
Soares et al. (2021)	d = 120 mm	—	—	100 copies	60
Xiong et al. (2020)	d = 81 mm	—	—	10 copies/μL	40
Dou et al. (2017c)	—	—	—	3 copies	45
Wu et al. (2021)	82 × 84 mm	—	—	30 copies	80
Kim et al. (2018)	20 × 20 mm	—	—	1 fg/μL	30
Nguyen et al. (2020)	—	—	—	3–300 copies	30
Ramachandran et al. (2020)	—	—	—	10 copies/μL	35
This work	25 × 25 mm	$160	6.4 W	2.2 copies	40

## 4 Conclusion

In this study, we developed microfluidic paper-based analytical devices that are characterized by simple fabrication, low cost, and ease of use. The filter paper material not only serves as a medium for self-driven reagent transport but also provides a large specific surface area for the reaction. We also designed a power-efficient portable instrument system which includes temperature control and optical modules. Automatic image acquisition and circle identification programs have been implemented to improve real-time signal processing capabilities. Using this approach, we achieved rapid detection of artificially synthesized SARS-CoV-2 RNA templates containing ORF1ab, N, and E gene fragments. Compared to conventional assays, reagent consumption was reduced by 89%, and the method demonstrated fine repeatability and sensitivity. The approach can be extended to molecular diagnosis and biochemical analysis; it may potentially improve the efficiency of large-scale infectious disease screening. In the next stage of our research, we plan to incorporate cloud computing and smartphones to enable mobile analytics.

## Data Availability

The raw data supporting the conclusions of this article will be made available by the authors, without undue reservation.

## References

[B1] Alteri Claudia Cento V. Antonello M. Colagrossi L. Merli M. Ughi N. (2020). Detection and quantification of SARS-CoV-2 by droplet digital PCR in real-time PCR negative nasopharyngeal swabs from suspected COVID-19 patients. PloS one 15 (9), e0236311. 10.1371/journal.pone.0236311 32898153 PMC7478621

[B2] Chan J. Yip C. C. Y. To K. K. W. Tang T. H. C. Wong S. C. Y. Leung K. H. (2020). Improved molecular diagnosis of COVID-19 by the novel, highly sensitive and specific COVID-19-RdRp/Hel real-time reverse transcription-PCR assay validated *in vitro* and with clinical specimens. J. Clin. Microbiol. 58 (5), e00310–e00320. 10.1128/jcm.00310-20 32132196 PMC7180250

[B3] Chi Junjie Shao C. Du X. Liu H. Gu Z. (2018). Generating microdroplet array on photonic pseudo-paper for absolute quantification of nucleic acids. ACS Appl. Mater. interfaces 10 (45), 39144–39150. 10.1021/acsami.8b11552 30335348

[B4] Dou Maowei Sanjay S. T. Dominguez D. C. Liu P. Xu F. Li X. (2017a). Multiplexed instrument-free meningitis diagnosis on a polymer/paper hybrid microfluidic biochip. Biosens. Bioelectron. 87, 865–873. 10.1016/j.bios.2016.09.033 27657849 PMC5125860

[B5] Dou Maowei Sanjay S. T. Dominguez D. C. Liu P. Xu F. Li X. (2017c). Multiplexed instrument-free meningitis diagnosis on a polymer/paper hybrid microfluidic biochip. Biosens. Bioelectron. 87, 865–873. 10.1016/j.bios.2016.09.033 27657849 PMC5125860

[B6] Dou Maowei Sanjay S. T. Dominguez D. C. Zhan S. Li X. (2017b). A paper/polymer hybrid CD-like microfluidic SpinChip integrated with DNA-functionalized graphene oxide nanosensors for multiplex qLAMP detection. Chem. Commun. 53 (79), 10886–10889. 10.1039/c7cc03246c PMC5626606 28703226

[B7] Garg Jaya Singh V. Pandey P. Verma A. Sen M. Das A. (2021). Evaluation of sample pooling for diagnosis of Covid‐19 by real time‐PCR: A resource‐saving combat strategy. J. Med. virology 93 (3), 1526–1531. 10.1002/jmv.26475 32869865

[B8] Hui Qingxin Pan Yuwei Yang Zhugen (2020). Paper-based devices for rapid diagnostics and testing sewage for early warning of COVID-19 outbreak. Case Stud. Chem. Environ. Eng. 2, 100064. 10.1016/j.cscee.2020.100064 PMC7700740 38620545

[B9] Jia Yuan Sun H. Dong H. Wang C. Lin X. Dong D. (2020). Scalable and parallelized biochemical assays in paper devices integrated with a programmable binary valve matrix. Sensors Actuators B Chem. 321, 128466. 10.1016/j.snb.2020.128466

[B10] Kim Jae-Heon Yoo I. S. An J. H. Kim S. (2018). A novel paper-plastic hybrid device for the simultaneous loop-mediated isothermal amplification and detection of DNA. Mater. Lett. 214, 243–246. 10.1016/j.matlet.2017.12.030

[B11] Krsak Martin Johnson Steven C. Poeschla Eric M. (2020). COVID-19 serosurveillance may facilitate return-to-work decisions. Am. J. Trop. Med. Hyg. 102 (6), 1189–1190. 10.4269/ajtmh.20-0302 32329432 PMC7253118

[B12] Lin Chengxiong Wang Y. Huang Z. Guo Y. Wu W. (2022). Low cost three-dimensional programmed mini-pump used in PCR. Micromachines 13 (5), 772. 10.3390/mi13050772 35630239 PMC9143699

[B13] Martinez Andres W. Phillips S. T. Whitesides G. M. Carrilho E. (2010). Diagnostics for the developing world: Microfluidic paper-based analytical devices. Anal. Chem. 3, 3–10. 10.1021/ac9013989 20000334

[B14] McConnell Witkowska Weronika W., (2021). Paper microfluidic implementation of loop mediated isothermal amplification for early diagnosis of hepatitis C virus. Nat. Commun. 12 (1), 1–8. 10.1038/s41467-021-27076-z 34848705 PMC8632961

[B15] Nguyen Long T. Smith Brianna M. Jain Piyush K. (2020). Enhancement of trans-cleavage activity of Cas12a with engineered crRNA enables amplified nucleic acid detection. Nat. Commun. 11 (1), 4906. 10.1038/s41467-020-18615-1 32999292 PMC7528031

[B16] Pekosz Andrew Parvu V. Li M. Andrews J. C. Manabe Y. C. Kodsi S. (2021). Antigen-based testing but not real-time polymerase chain reaction correlates with severe acute respiratory syndrome coronavirus 2 viral culture. Clin. Infect. Dis. 73 (9), e2861–e2866. 10.1093/cid/ciaa1706 33479756 PMC7929138

[B17] Piana Andrea Colucci M. E. Valeriani F. Marcolongo A. Sotgiu G. Pasquarella C. (2021). Monitoring COVID-19 transmission risks by quantitative real-time PCR tracing of droplets in hospital and living environments. MSphere 6 (1), 010700–e1120. 10.1128/msphere.01070-20 PMC7845593 33408231

[B18] Ramachandran Ashwin Huyke D. A. Sharma E. Sahoo M. K. Huang C. Banaei N. (2020). Electric field-driven microfluidics for rapid CRISPR-based diagnostics and its application to detection of SARS-CoV-2. Proc. Natl. Acad. Sci. 117 (47), 29518–29525. 10.1073/pnas.2010254117 33148808 PMC7703567

[B19] Reboud Julien Xu G. Garrett A. Adriko M. Yang Z. Tukahebwa E. M. (2019). Paper-based microfluidics for DNA diagnostics of malaria in low resource underserved rural communities. Proc. Natl. Acad. Sci. 116 (11), 4834–4842. 10.1073/pnas.1812296116 30782834 PMC6421471

[B20] Seok Youngung Joung H. A. Byun J. Y. Jeon H. S. Shin S. J. Kim S. (2017). A paper-based device for performing loop-mediated isothermal amplification with real-time simultaneous detection of multiple DNA targets. Theranostics 7 (8), 2220–2230. 10.7150/thno.18675 28740546 PMC5505055

[B21] Soares Ruben R. G. Akhtar A. S. Pinto I. F. Lapins N. Barrett D. Sandh G. (2021). Sample-to-answer COVID-19 nucleic acid testing using a low-cost centrifugal microfluidic platform with bead-based signal enhancement and smartphone read-out. Lab a Chip 21 (15), 2932–2944. 10.1039/d1lc00266j 34114589

[B22] Sreejith Kamalalayam Rajan Umer M. Dirr L. Bailly B. Guillon P. von Itzstein M. (2021). A portable device for LAMP based detection of SARS-CoV-2. Micromachines 12 (10), 1151. 10.3390/mi12101151 34683202 PMC8538454

[B23] Sun Hao Jia Y. Dong H. Dong D. Zheng J. (2020). Combining additive manufacturing with microfluidics: An emerging method for developing novel organs-on-chips. Curr. Opin. Chem. Eng. 28, 1–9. 10.1016/j.coche.2019.10.006

[B24] Sun Hao Jia Y. Dong H. Fan L. (2019). Graphene oxide nanosheets coupled with paper microfluidics for enhanced on-site airborne trace metal detection. Microsystems Nanoeng. 5 (1), 4–12. 10.1038/s41378-018-0044-z PMC6369225 31057931

[B25] Sun Hao Jiang Q. Huang Y. Mo J. Xie W. Dong H. (2023). Integrated smart analytics of nucleic acid amplification tests via paper microfluidics and deep learning in cloud computing. Biomed. Signal Process. Control 83, 104721. 10.1016/j.bspc.2023.104721

[B26] Sun Hao Xiong L. Huang Y. Chen X. Yu Y. Ye S. (2021). AI-Aided on-chip nucleic acid assay for smart diagnosis of infectious disease. Fundam. Res. 2, 476–486. 10.1016/j.fmre.2021.12.005 PMC8712671 40477452

[B27] Tahamtan Alireza Ardebili Abdollah (2020). Real-time RT-PCR in COVID-19 detection: Issues affecting the results. Expert Rev. Mol. diagnostics 20 (5), 453–454. 10.1080/14737159.2020.1757437 PMC7189409 32297805

[B28] Tarim E. A. Anil Inevi M. Ozkan I. Kecili S. Bilgi E. Baslar M. S. (2023a). Microfluidic-based technologies for diagnosis, prevention, and treatment of COVID-19: Recent advances and future directions. Biomed. Microdevices 25 (2), 10. 10.1007/s10544-023-00649-z 36913137 PMC10009869

[B29] Tarim E. A. Karakuzu B. Oksuz C. Sarigil O. Kizilkaya M. Al-Ruweidi M. K. A. A. (2021). Microfluidic-based virus detection methods for respiratory diseases. Emergent Mater. 4, 143–168. 10.1007/s42247-021-00169-7 33786415 PMC7992628

[B30] Tarim E. A. Oksuz C. Karakuzu B. Appak O. Sayiner A. A. Tekin H. C. (2023b). Electromechanical RT-LAMP device for portable SARS-CoV-2 detection. Talanta 254, 124190. 10.1016/j.talanta.2022.124190 36521325 PMC9733968

[B31] Thompson Dorian Lei Yu (2020). Mini review: Recent progress in RT-LAMP enabled COVID-19 detection. Sensors Actuators Rep. 2 (1), 100017. 10.1016/j.snr.2020.100017 PMC7428436 35047828

[B32] Tomita Norihiro Mori Y. Kanda H. Notomi T. (2008). Loop-mediated isothermal amplification (LAMP) of gene sequences and simple visual detection of products. Nat. Protoc. 3 (5), 877–882. 10.1038/nprot.2008.57 18451795

[B33] Wei Xiaofeng Tian T. Jia S. Zhu Z. Ma Y. Sun J. (2016). Microfluidic distance readout sweet hydrogel integrated paper-based analytical device (μDiSH-PAD) for visual quantitative point-of-care testing. Anal. Chem. 88 (4), 2345–2352. 10.1021/acs.analchem.5b04294 26765320

[B34] Whitesides George M. (2006). The origins and the future of microfluidics. nature 442 (7101), 368–373. 10.1038/nature05058 16871203

[B35] World Bank Group (2020). Poverty and shared prosperity 2020. Available: https://www.worldbank.org/en/publication/poverty -and-shared-prosperity.

[B36] World Health Organization (2022). Disease outbreak news (DONs) . https://www.who.int/emergencies/disease-outbreak-news (Accessed July 15, 2022).

[B37] Wu Hui Qian S. Peng C. Wang X. Wang T. Zhong X. (2021). Rotary valve-assisted fluidic system coupling with CRISPR/Cas12a for fully integrated nucleic acid detection. ACS sensors 6 (11), 4048–4056. 10.1021/acssensors.1c01468 34665590

[B38] Xiong Huiwen Ye X. Li Y. Wang L. Zhang J. Fang X. (2020). Rapid differential diagnosis of seven human respiratory coronaviruses based on centrifugal microfluidic nucleic acid assay. Anal. Chem. 92 (21), 14297–14302. 10.1021/acs.analchem.0c03364 33073982

[B39] Xu Gaolian Nolder D. Reboud J. Oguike M. C. van Schalkwyk D. A. Sutherland C. J. (2016). Paper‐origami‐based multiplexed malaria diagnostics from whole blood. Angew. Chem. 128 (49), 15476–15479. 10.1002/ange.201606060 PMC5132111 27554333

[B40] Yang Ruey-Jen Tseng C. C. Ju W. J. Fu L. M. Syu M. P. (2018a). Integrated microfluidic paper-based system for determination of whole blood albumin. Sensors Actuators B Chem. 273, 1091–1097. 10.1016/j.snb.2018.07.010

[B41] Yang Zhugen Xu G. Reboud J. Ali S. A. Kaur G. McGiven J. (2018b). Rapid veterinary diagnosis of bovine reproductive infectious diseases from semen using paper-origami DNA microfluidics. ACS sensors 3 (2), 403–409. 10.1021/acssensors.7b00825 29322764

[B42] Yao Yuhan Chen X. Zhang X. Liu Q. Zhu J. Zhao W. (2020). Rapid detection of influenza virus subtypes based on an integrated centrifugal disc. ACS sensors 5 (5), 1354–1362. 10.1021/acssensors.9b02595 32248677

[B43] Yao Yuhan Zhao N. Jing W. Liu Q. Lu H. Zhao W. (2021). A self-powered rapid loading microfluidic chip for vector-borne viruses detection using RT-LAMP. Sensors Actuators B Chem. 333, 129521. 10.1016/j.snb.2021.129521

[B44] Zhang Mimi Liu J. Shen Z. Liu Y. Song Y. Liang Y. (2021). A newly developed paper embedded microchip based on LAMP for rapid multiple detections of foodborne pathogens. BMC Microbiol. 21 (1), 197–213. 10.1186/s12866-021-02223-0 34182947 PMC8240391

