# Supplementary material for: A portable system for economical nucleic acid amplification testing
Source: Front Bioeng Biotechnol. 2023 Aug 2;11:1214624. doi: 10.3389/fbioe.2023.1214624 (PMC10436208; doi:10.3389/fbioe.2023.1214624)
Supplement: Supplementary file 1 [file DataSheet1.pdf]

Supplementary Material

A Portable System for Economic Nucleic Acid Amplification Test

Hui Dong[1,2*], Jin Mo[1,2], Yongjian Yu[1,2], Wantao Xie[1,2*], Jianping Zheng[3], Chao Jia[1,2*]

[1]School of Mechanical Engineering and Automation, Fuzhou University, Fuzhou, 350116, China

[2]Fujian Provincial Collaborative Innovation Center of High-End Equipment Manufacturing, Fuzhou, 350116, China

[3]Fujian Provincial Hospital, Fuzhou, Fujian 350001, China

*** Correspondence:** Corresponding Author: hdong@fzu.edu.cn; wt.xie@qq.com; chaojia@fzu.edu.cn

1 Supplementary Figures and Tables

1.1 Supplementary Figures

The chip channels were designed with two centrally symmetric rectangular patterns, including four sample inlets/outlets and two circular paper-based detection areas. The sample inlets/outlets were circular with a diameter of 1.5mm, while the detection areas were circular with a diameter of 2.5mm. The connecting channel section had a width of 1mm. The distance between the centers of the two detection areas was 5mm. The channel had openings at both ends to allow air flow. When reagents were introduced at one end, they entered the channel under gravity and capillary forces, displacing the air and exiting from the other end. Upon contact with the paper-based reaction area, the reagents were rapidly absorbed and stored.

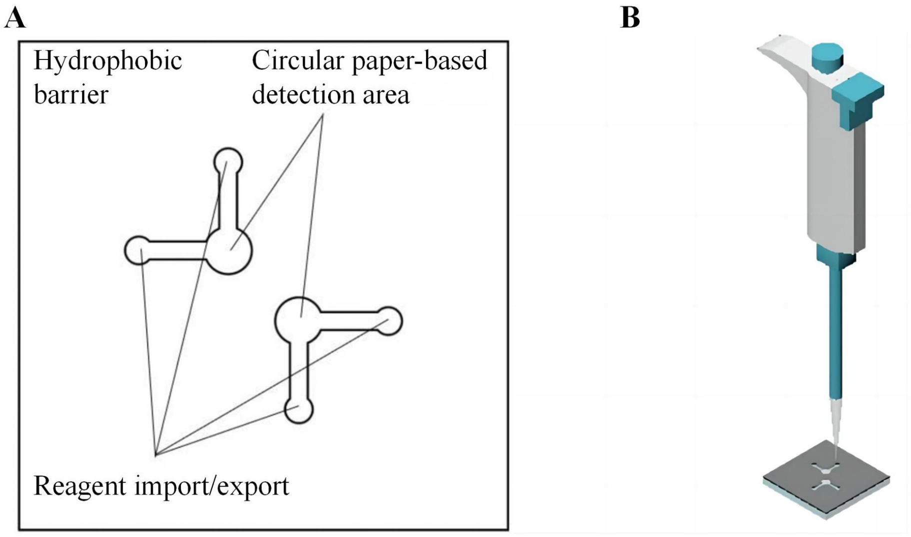

Supplementary Figure 1. (**A**) Chip channel design. (**B**) Schematic diagram of sample loading.

As a reference, we measured the temperature of MCH via a thermocouple thermometer under the same condition (voltage 1.7 V, current 2.4 A). The result showed that the edge temperature of the MCH was 69.9 ℃, and the center temperature of the chip was 64.9 ℃, which were basically consistent with those obtained from the infrared thermal imager.

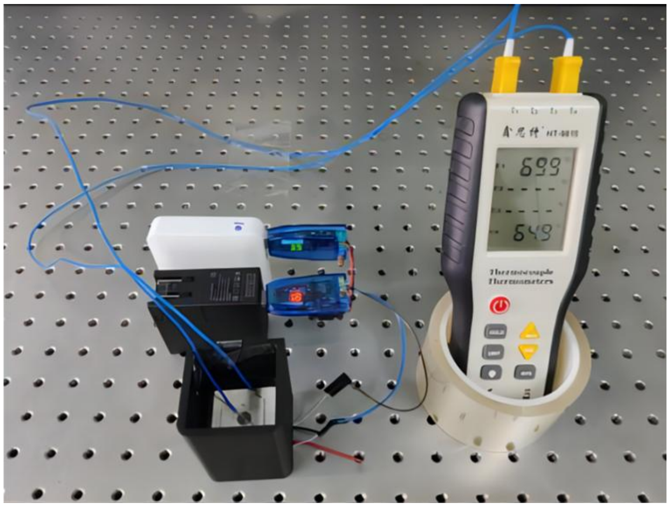

Supplementary Figure 2. Reference measurement of chip temperature via a thermocouple thermometer.

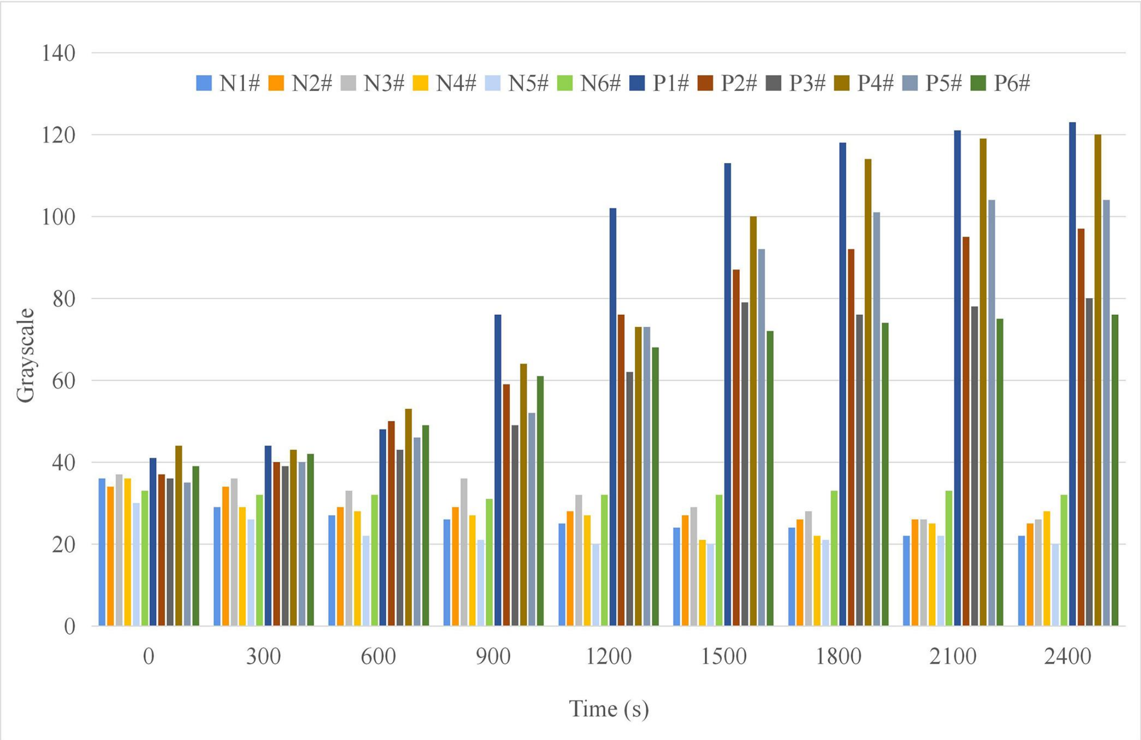

Supplementary Figure 3. Analysis of positive samples and negative samples in a bar chart. N1#-N6#: negative samples, P1#-P6#: positive samples.

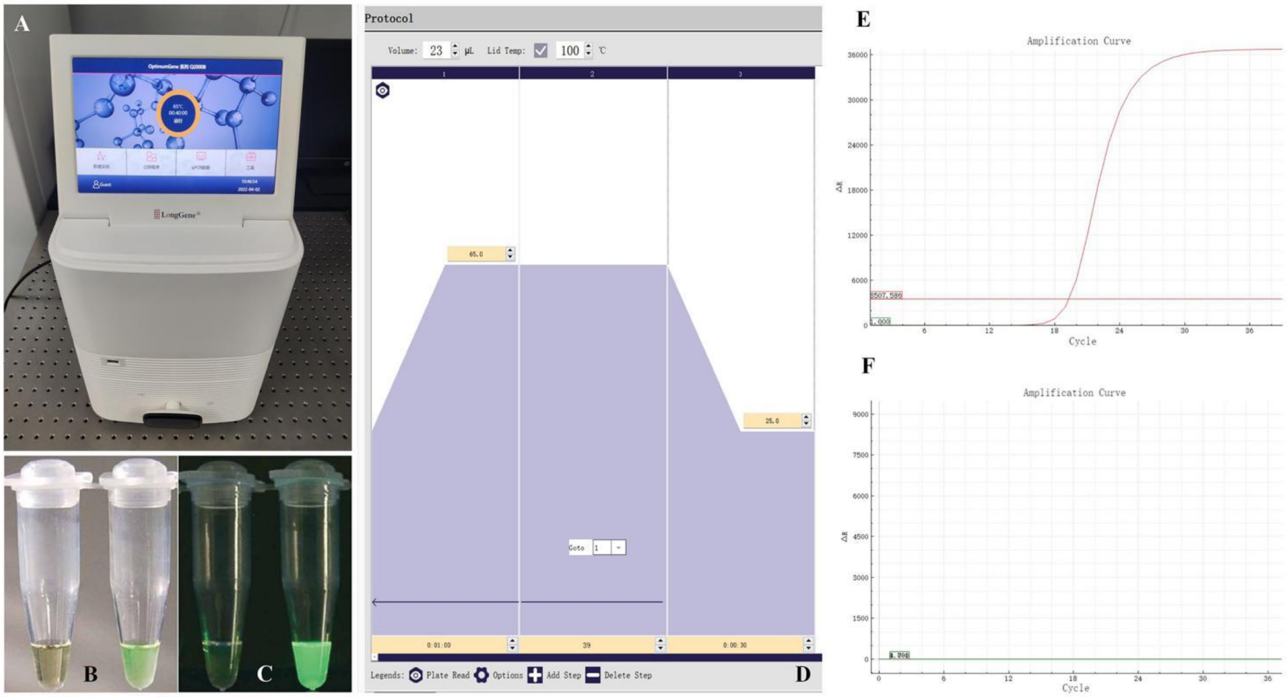

Supplementary Figure 4. Tube-based experiment. (**A**) LongGene® Q2000B fluorescence quantitative PCR instrument. (**B**) Negative control (left) and positive sample (right) after reaction under daylight. (**C**) Negative control (left) and positive sample (right) after reaction in a dark environment. (**D**) Settings of instrument in the experiment. 1 minute per cycle, with a total of 40 cycles. (**E**) Amplification Curve of positive sample exported from the instrument. (**F**) Amplification Curve of negative control exported from the instrument.

1.2 Supplementary Tables

Supplementary Table 1

Cost of system components and each paper-based chip

**Cost of system components**			**Cost of each paper-based chip**			
**Component**	**Price ($)**	**Number**	**Material**	**Price ($)**	**Consumption**	**Cost ($)**
Shell	1.33	1	Glass	525.10/m^2	400mm^2	0.2100
Blue light LED + Cooler	0.7	1	Opaque PVC	8.79/m^2	1600mm^2	0.0020
Filter	28.01	2	PMMA	7.70/m^2	400mm^2	0.0004
Porcelain heater	2.24	1	Thermal PVC film	2.40/m^2	400mm^2	0.0001
Lithium battery	13.86	2	Filter paper	157.21/m^2	100mm^2	0.0022
Cooling fan	0.42	1				

USB voltage regulator	2.24	3		
CMOS HD camera	62.73	1		
**Total**	**157.88**		**Total**	**0.2148**

Supplementary Table 2

Materials employed in the chip

Layer	Number	Material
Detection grid	1	Pressure Sensitive Black PVC Adhesive
Adhesive film	2	Transparent PET double-sided tape
Cover film	1	Thermal PVC film
Paper-based reaction unit	2	Whatman Grade 1 filter paper
Support frame	1	Transparent PET double-sided tape
Hydrophobic frame	1	Pressure Sensitive Black PVC Adhesive
Substrate	1	High-purity quartz glass

Supplementary Table3

Workflow of the experiment

1	Place the sealing adhesive, chip, and ice pack on a clean, new A4 paper in the cabinet.
2	Check the number of pipettes and tips to ensure an adequate supply.
3	After arranging the experimental equipment neatly, turn on the UV lamp inside the workspace and expose it to light for 15 minutes. Ensure that no one is present in the room or cover the exposed area with aluminum foil.
4	Take out the LAMP reagent kit (including premix and positive control) from the refrigerator.
5	Spray alcohol on the ventilation vent of the workspace and the LAMP reagent kit, then wipe them clean.
6	Spray a small amount of RNase and DNase cleaning solution and gently wipe all surfaces inside the cabinet with a lint-free cloth.
7	Take out the reaction buffer and positive sample extraction tubes from the reagent kit and place them on the ice pack. Allow the reagents to thaw naturally before further preparation.
8	Prepare the LAMP reagent mixture according to the instructions.
9	Hold the prepared LAMP reagent in your hand and shake it up and down several times to ensure thorough mixing with the lyophilized powder on the tube cap.

10	Centrifuge the LAMP reagent (approximately 18μL) at 2000rpm for 20 seconds.
11	Retrieve the LAMP reagent tube and wipe the external surface with alcohol, RNase cleaning solution, and DNase cleaning solution in sequence.
12	Adjust the pipette volume to 2.2μL and open the cap of the reagent tube, placing it on top of the tube.
13	Expel the air from the pipette before use and ensure deep insertion into the vial, aspirating 2.2μL at once.
14	Quickly and gently dispense the 2.2μL of reagent into the chip inlet and observe if the sample has entered the reaction unit.
15	After using the reagent, immediately close the cap tightly and place it above the ice pack.
16	After using all the reagents, wrap them in aluminum foil and return them to the refrigerator.
17	When sealing the chip, press the edges firmly with tweezers.
18	Close the workspace.
19	Place the chip on top of the ceramic heating plate of the integrated self-driven microfluidic system and assemble the system in the correct order.
20	Set the experimental parameters, start the microfluidic system, and begin the experiment.
21	After completing the experiment, seal the discarded samples and handle them regularly.
